# Polymorphisms in the TGFB1 and FOXP3 genes are associated with the presence of antinuclear antibodies in chronic hepatitis C

**DOI:** 10.1016/j.heliyon.2020.e04524

**Published:** 2020-07-22

**Authors:** Geison Luiz Costa de Castro, Carlos David A. Bichara, Angélica Menezes Santiago, William Botelho de Brito, Leonn Mendes Soares Pereira, Tuane Carolina Ferreira Moura, Ednelza da Silva Graça Amoras, Mauro Sérgio Moura de Araújo, Simone Regina Souza da Silva Conde, Maria Alice Freitas Queiroz, Ricardo Ishak, Antonio Carlos Rosário Vallinoto

**Affiliations:** a Laboratório de Virologia, Instituto de Ciências Biológicas, Universidade Federal do Pará, Belém, PA, Brazil; b Hospital Universitário João de Barros Barreto, Universidade Federal do Pará, Belém, PA, Brazil

**Keywords:** Microbiology, Immunology, Virology, Immune response, Viruses, Infectious disease, Chronic hepatitis C, Ana, TGF-β1, FOXP3, Polymorphisms

## Abstract

Chronic infection with Hepacivirus C (HCV) can lead to the occurrence of antinuclear antibodies (ANAs) and changes in cytokine profiles that can be similar to autoimmune diseases. The aim of the study was to identify polymorphisms in important mediators of the immune response in association with ANAs, which could contribute to the development of autoimmunity in hepatitis C. The study included 87 patients with chronic hepatitis C who were evaluated for the presence of ANA (indirect immunofluorescence) and for polymorphisms in the *FOXP3*, *IFNG*, *IL6*, *IL8*, *IL10*, *MBL2*, *CRP*, *TGFΒ1* and *TNFA* genes (real-time PCR). Of the patients evaluated, 17 (19.54%) had ANA reactivity. The G allele of the *FOXP3* rs2232365 polymorphism was more frequent in ANA-positive women (p = 0.0231; OR = 3,285). The C allele of the *TGFΒ1* rs1800469 polymorphism was associated with ANA production (p = 0.0169; OR = 2.88). The results suggest that polymorphisms in genes related to immunological regulation may be associated with mechanisms that lead to the emergence of autoantibodies in the context of chronic Hepacivirus C infection.

## 1 Introduction

Hepacivirus C infection affects approximately 71 million people worldwide, leading to the death of more than 400,000 people each year due to complications such as liver cirrhosis and hepatocellular carcinoma (Ringehan et al., 2017; WHO, 2017). In addition, several studies have shown that in some cases of chronic hepatitis C, non-organ-specific autoantibodies, such as antinuclear antibodies (ANAs), which can occur in up to 40% of cases, are produced (Acay et al., 2015; Narciso-Schiavon and Schiavon, 2015; Navarta et al., 2018). However, the mechanisms that link infection to autoimmune processes are not well established.

In chronic hepatitis C, there are changes in the expression profiles of mediators of the immune response of several interleukins (ILs), such as IL-6, IL-8, and IL-10; interferon γ (IFN-γ); growth transformation factor β (TGF-β); C-reactive protein (CRP), and tumor necrosis factor α (TNF-α), factors linked to immunological tolerance, such as forkhead box P3 (FOXP3) (Amoras et al., 2016; de Souza-Cruz et al., 2016; Moura et al., 2019; R-Viso et al., 2010; Sofian et al., 2012). Single nucleotide polymorphisms (SNPs) can alter the expression levels or functions of these factors, leading to a predisposition to the development or evolution of liver diseases (Moura et al., 2017; Pereira et al., 2018).

Thus, there may be an intersection between genetic factors that promote changes in cytokine production and the development of autoantibodies in chronic hepatitis C (Atfy et al., 2009; Slight-Webb et al., 2016; Torell et al., 2019). In this sense, the objective of the present study was to identify polymorphisms in important mediators of the immune response (FOXP3, IFNG, IL6, IL8, IL10, MBL2, CRP, TGFΒ1 and TNFA) in association with ANAs, which could contribute to the development of autoimmunity in hepatitis C.

## 2 Materials and methods

### 2.1 Study population and ethical aspects

The study evaluated 87 patients with chronic hepatitis C from the Santa Casa de Misericórdia do Pará Foundation and at the João de Barros Barreto University Hospital of the Federal University of Pará.

The inclusion criteria consisted of individuals aged 18 years or older, of both sexes, and positivity for anti-HCV and HCV-RNA. The study excluded individuals who did not meet the requirements stipulated above, patients with previous diagnosis of autoimmune hepatitis, those patients coinfected with hepatitis B virus (HBV) and/or HIV-1, and patients who used or were using specific antiviral therapy against HBV or HCV.

This study was approved by the Research Ethics Committee of the Santa Casa de Misericórdia do Pará Foundation (protocol 772.782/2014) and by the Research Ethics Committee of the João de Barros Barreto University Hospital (protocols 962.537/2015 and 2.165.948/2017). All patients who agreed to participate in the study signed an informed consent form.

### 2.2 ANA detection

Qualitative ANA research was performed using the direct immunofluorescence method with the Antinuclear Antibody/ANA/Hep-2 VIRGO kit (Hemagen Diagnostics, USA) in plasma samples, according to the manufacturer's specifications. Samples with positive results were considered to be those with reactivity in titration 1/80, as recommended by the IV Brazilian Consensus for Research of Autoantibodies in HEp-2 Cells (Francescantonio et al., 2013).

### 2.3 Sampling

Blood samples (5 mL) were collected using a vacuum collection tube containing ethylenediaminetetraacetic acid (EDTA) as an anticoagulant. Then, the samples were separated into cell mass and plasma, which were stored at -20 °C until use.

### 2.4 DNA extraction

The extraction of total DNA from peripheral blood cells was performed according to the protocol of Cigliero et al. (2011). In this method, the cell lysis was performed using 0.2 M Na-Acetate pH 7.0, 10% SDS and Proteinase K. The protein precipitation was performed using a Phenol/chloroform/Isoamyl alcohol (25:24:1 v/v/v) solution. On the final stages, DNA precipitation was performed with 100% ethanol and hydration with sterile water.

### 2.5 Genotyping

The genotyping of polymorphisms in the *FOXP3* genes (rs2232365, rs3761548, rs3761549), *IFNG* (rs2430561), *IL6* (rs1800795), *IL8* (rs4073), *IL10* (rs1800896), *MBL2* (rs1800450, rs1800451, rs2130457, CR2130727) (rs1800469) and *TNFA* (rs1800629) was performed by real-time PCR using StepOne PLUS Sequence Detector equipment (Applied Biosystems, Foster City, CA, USA). Predesigned and customized TaqMan® SNP Genotyping Assay assays were used (Table 1). For each reaction, 7 μL of distilled water, 10 μL of TaqMan® Universal PCR Master Mix (2X), 1 μL of TaqMan® Assay (20X) and 2 μL of extracted DNA were used, totaling 20 μL of final volume. The following temperature cycles were used in the amplification: 60 °C for 30 seconds, followed by 95 °C for 10 minutes, 50 cycles of 92 °C for 30 seconds and 1 cycle at 60 °C for 1 minute and 30 seconds.

**Table 1:** Customized tests for the TaqMan® panel used in the study.

Gene	Polymorphism	Mutation	Region	Assay
FOXP3	rs2232365	A > G	Intron	C_15942641_10
FOXP3	rs3761548	C > A	Intron	C_27476877_10
FOXP3	rs3761549	C > T	Intron	C_27058744_10
IL6	rs1800795	G > C	Intron	C_1839697_20
IL8	rs4073	A > T	2 KB upstream	C_11748116_10
IL10	rs1800896	A > G	2 KB upstream	C___1747360_10
IFNG	rs2430561	T > A	Intron	C_8708473_10
MBL2	rs1800450	G > A	Missense	C_12336609_20
MBL2	rs1800451	G > A	Missense	C2336608__20
MBL2	rs5030737	C > T	Missense	C_2336610_20
CRP	rs2794521	T > C	2 KB upstream	C_318207_20
TGFB1	rs1800469	C > T	2 KB upstream	Customized
TNFA	rs1800629	G > A	2 KB upstream	C_7514879_10

### 2.6 Statistical analysis

For the evaluation of Hardy-Weinberg equilibrium, the chi-square test was used, specifically for the polymorphisms in the *FOXP3* gene, and the balance was calculated only for the female gender. The intergroup allelic frequencies were estimated by the chi-square and Fisher's exact tests. The odds-ratio calculation was used to infer the association of alleles with the presence of ANA. For statistical tests, BioEstat software version 5.0 (Ayres et al., 2008) was used with a significance value of 95% (p ≤ 0.05). Heatmap grouping plots were proposed based on sex, the presence of ANA and the polymorphic variants investigated.

## 3 Results

**Figure 1:**
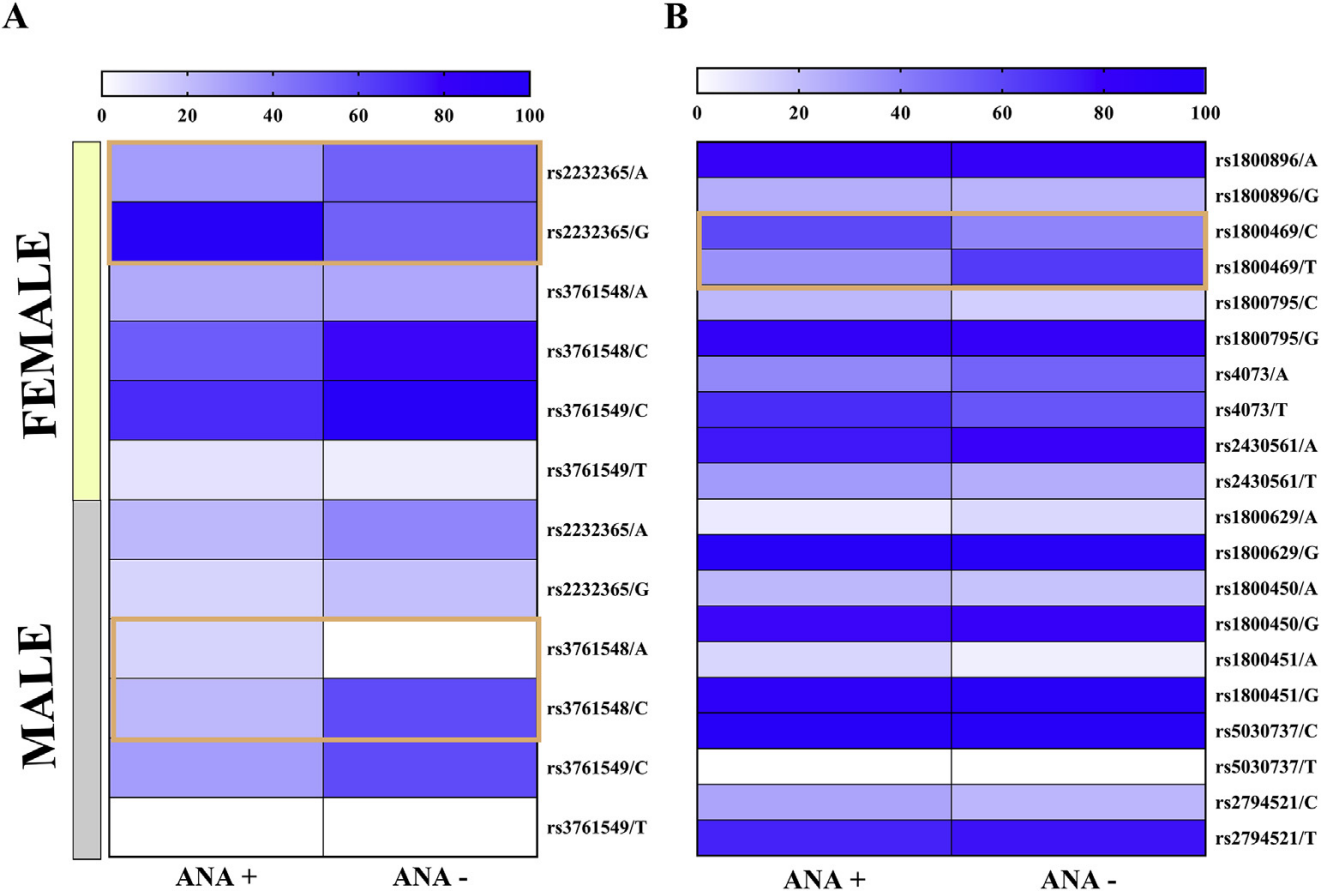
Heatmap showing the frequencies of the polymorphisms analyzed in the groups of ANA-positive and ANA-negative patients (A) frequencies of the X-linked polymorphisms and (B) frequencies of the autossomic polymorphisms. The blue boxes highlight the polymorphisms with significative frequencies in ANA-positive patients.

The prevalence of ANA in patients with chronic hepatitis C was 19.54%. All polymorphisms were in Hardy-Weinberg equilibrium; when the allele frequencies of the intergroup polymorphisms were compared, significant differences were observed in the distribution of the variants in the *FOXP3* and *TGF-β1* genes (Figure 1; Table 2).

**Table 2:** Allele frequencies of the polymorphisms analyzed in the groups of ANA-positive and ANA-negative patients.

SNP	N	ANA positive	ANA negative	*p*	*Odds ratio*	95% CI
**Anti-inflammatory and regulatory profile**						
***FOXP3***						
rs2232365						
Female	40					
A		7 (0.39)	31 (0.50)	0.0231	3.285	1.23–8.78
G		23 (0.61)	31 (0.50)			
Male	46					
A		5 (0.63)	24 (0.63)	0.9977		
G		3 (0.37)	14 (0.37)			
rs3761548						
Female	41					
A		6 (0.33)	18 (0.28)	0.7708		
C		12 (0.67)	46 (0.72)			
Male	46					
A		3 (0.38)	3 (0.08)	0.0559	7.00	1.10–44.72
C		5 (0.62)	35 (0.92)			
rs3761549						
Female	41					
C		16 (0.44)	58 (0.45)	0.9889		
T		2 (0.56)	6 (0.55)			
Male	46					
C		7 (01.00)	35 (0.46)	1.0000		
T		0	3 (0.54)			
***IL10***						
rs1800896	87					
A		26 (0.76)	109 (0.78)	0.9559		
G		8 (0.24)	31 (0.22)			
***TGFB1***						
rs1800469	83					
C		19 (0.63)	51 (0.38)	0.0169	2.88	1.27–6.53
T		11 (0.37)	85 (0.63)			
**Proinflammatory profile**						
***IL6***						
rs1800795	86					
C		7 (0.21)	20 (0.14)	0.5405		
G		27 (0.79)	118 (0.86)			
***IL8***						
rs4073	86					
A		12 (0.35)	66 (0.48)	0.2617		
T		22 (0.65)	72 (0.52)			
***IFNG***						
rs2430561	86					
A		24 (0.71)	104 (0.75)	0.7248		
T		10 (0.29)	34 (0.25)			
***TNF-α***						
rs1800629	87					
A		2 (0.06)	16 (0.11)	0.5317		
G		32 (0.94)	124 (0.89)			
***MBL2***						
rs1800450	82					
A		7 (0.22)	25 (0.19)	0.8987		
G		25 (0.78)	107 (0.81)			
rs1800451	81					
A		4 (0.13)	7 (0.5)	0.2298		
G		28 (0.87)	123 (0.95)			
rs5030737	82					
C		32 (1.00)	131 (0.99)	1,0000		
T		0	1 (0,01)			
***PrtCR***						
rs2794521	81					
C		9 (0.28)	30 (0.23)	0.7132		
T		23 (0.72)	100 (0.77)			

In *FOXP3*, the G allele of the rs2232365 polymorphism was more frequent in the HCV-positive women than the controls (p = 0.0231) and was associated with a relative increase in the risk for ANA production of approximately 27% (OR: 3,285; CI: 1.23–8.78). The A allele of the rs3761548 polymorphism occurred 7 times more frequently in the ANA-positive men than the control men, with an associated risk increase of approximately 30% in the development of autoantibodies (p = 0.0559; OR = 7.00; CI = 1.10–44.72) (Table 2).

Variant C of the *TGF-β1* polymorphism rs1800469 was more frequent in the patients with ANA than the controls (p = 0.0169), indicating it is a risk factor for the emergence of ANA in patients with chronic hepatitis C (OR = 2.88; CI = 1.27–6.53).

## 4 Discussion

The link between Hepacivirus C and the development of autoimmunity is evidenced by the detection of autoantibodies in a high number of patients with chronic hepatitis C (Narciso-Schiavon and Schiavon, 2015) and by the high prevalence of autoimmune diseases in these patients (Younossi et al., 2013). Roughan et al. (2012) showed that in chronic infection, Hepacivirus C can promote the polyclonal expansion of autoreactive B lymphocytes that escape the mechanisms of immunological tolerance, which results in the excessive production of autoantibodies.

Changes in the nuclear and cytoplasmic molecules of infected cells can be recognized and treated as targets of the autoimmune response, compromising peripheral tolerance mechanisms and contributing to the induction of autoimmunity, which in systemic lupus erythematosus (SLE) is mainly marked by ANA induction (Baumann et al., 2002; Burbano et al., 2018).

ANAs are immunoglobulins that have specificity for different structural and functional components of cells, thus mediating the pathological processes of inflammation and the consequent tissue damage (Agmon-Levin et al., 2014; Tan, 2014). In contrast, TGF-β1 is a fundamental immunoregulatory cytokine that maintains immunological tolerance against self-antigens (Kelly et al., 2017). In the context of chronic hepatitis C, high concentrations of TGF-β1 are observed compared to those in healthy individuals (Nelson et al., 1997), mainly due to the interference of the virus in the signaling pathways related to the expression of this cytokine (Chusri et al., 2016). In addition, this protein plays an important role in inducing hepatic fibrosis through activation of hepatic stellate cells (Zhou et al., 2014).

Polymorphisms in the *TGFB1* gene can alter the circulating levels of cytokines. Studies have shown that the C allele of the rs1800469 polymorphism decreases the mRNA expression of this gene (Grainger et al., 1999; Panek et al., 2014), possibly by altering the binding site of hypoxia-inducible factor 1A (HIF1A) and activator protein 1 (AP-1) (Shah et al., 2006). In the present study, the frequency of the C allele was associated with the risk of autoantibody production in patients with chronic hepatitis C; these results suggested that the negative regulation of the variant may influence the maintenance of immunological tolerance by negative feedback through TGF-β1.

In fact, TGF-β1 suppresses adaptive responses acting on different mechanisms of B and T cells, including potential autoreactive lymphocytes. This cytokine inhibits T cell function, interfering with the development of TCR signaling (Heath et al., 2000; Gorelik et al., 2002; Chen et al., 2003; Oh and Li, 2013). At the B cell level, TGF-β1 can interfere with the processes of cell differentiation and proliferation, as well as the synthesis of immunoglobulins and their class changes (Kehrl et al. 1986, 1991; Tamayo et al., 2018). In addition, TGF-β1 can induce the conversion of CD4+ T cells into Treg cells by inducing *FOXP3* expression (Fantini et al., 2004), as well as increasing the expression of other essential markers for the functioning of Treg cells, such as CD25, CD122, CTLA-4 and IL-2 (Zheng et al., 2014).

Similarly, polymorphisms in the *FOXP3* gene can also alter the expression levels of this gene (Oda et al., 2013). In the present study, the G alleles of the rs2232365 polymorphism and A allele of the rs3761548 polymorphism were associated with the risk of production of autoantibodies. Both alleles decrease *FOXP3* gene expression. The G allele (rs2232365) alters the binding site of the GATA3 factor and has been associated with a predisposition to autoimmune diseases (Song et al., 2013). The A allele (rs3761548) is associated with a reduction in the levels of gene expression due to the loss of the ability to bind to transcription factors such as E47 and C-Myb (Shen et al., 2010).

The FOXP3 protein is associated with the differentiation and function of Treg cells and, therefore, the maintenance of immune tolerance and the regulation of immune responses (Pereira et al., 2017). Homeostasis promotes the maintenance of tolerance by suppressing the activation, proliferation and effector functions of different cells of the immune system, including autoreactive lymphocytes (Arce-Sillas et al., 2016; Jung and Shin, 2016). Thus, changes in the expression pattern of FOXP3 lead to loss of action by Treg cells, resulting in increased damage caused by immune responses, which is common in autoimmune processes (Claassen et al., 2010; Jung and Shin, 2016).

The present study suggests that genetic factors linked to the regulation of FOXP3 and TGF-β1, in the context of chronic hepatitis C, can lead to functional changes in Treg cells also and result in a greater propensity for failures in suppressing the immune response and the mechanisms of tolerance to self-antigens. These changes may lead to more severe inflammation and the production of autoantibodies.

In the context of liver autoimmune diseases marked by the presence of ANA, the loss of tolerance in the liver results from the loss of inhibitory functions of the immune system that results mainly from the decrease in quantity and function of the Treg cells (Liberal et al., 2015). In patients with chronic HCV infection and ANA positivity, suppression of Treg cells was observed, with a reduction in cellular markers, including FOXP3 (Fouad et al., 2016).

In conclusion, the results showed that the *TGFΒ1* rs1800469 and *FOXP3* rs2232365 genetic variations, related to the reduction of gene expression, seem to influence the control of tolerance to self-antigens and contribute to the development of autoimmune manifestations in patients with chronic hepatitis C.

## Declarations

### Author contribution statement

Geison Luiz Costa de Castro: Conceived and designed the experiments; Performed the experiments; Wrote the paper.

Carlos David A. Bichara: Conceived and designed the experiments; Performed the experiments.

Angélica Menezes Santiago, William Botelho de Brito, Leonn Mendes Soares Pereira, Tuane Carolina Ferreira Moura: Performed the experiments.

Ednelza da Silva Graça Amoras, Mauro Sérgio Moura de Araújo, Simone Regina Souza da Silva Conde, Maria Alice Freitas Queiroz: Analyzed and interpreted the data.

Ricardo Ishak, Antonio Carlos Rosário Vallinoto: Conceived and designed the experiments; Contributed reagents, materials, analysis tools or data; Wrote the paper.

### Funding statement

This work was supported by the 10.13039/501100003593National Council for Scientific and Technological Development of Brazil (CNPQ# 480128/2013-8; #301869/2017-0) and the 10.13039/501100007382Federal University of Pará (PROPESP/PAPQ/2019).

### Competing interest statement

The authors declare no conflicts of interest.

### Additional information

No additional information is available for this paper.
